# Phytosphingosine disrupts dual-species oral biofilms and attenuates LPS-induced pro-inflammatory responses in human gingival fibroblasts

**DOI:** 10.1038/s41598-026-55714-3

**Published:** 2026-05-30

**Authors:** Nattaset Chamnanvinijchai, Nicha Techawatcharathep, Natnicha Ngerndee, Sittikorn Teerawongwiwat, Boonjira Anukul, Panitan Jumjitvi, Floris J. Bikker, Kamran Nazmi, Henderikus Pots, Watcharapong Panthong, Saharut Wongkaewkhiaw

**Affiliations:** 1 School of Dentistry, King Mongkut’s Institute of Technology Ladkrabang, Bangkok, Thailand; 2 Department of Oral Biochemistry, Academic Centre for Dentistry Amsterdam (ACTA), University of Amsterdam and Vrije Universiteit Amsterdam, Amsterdam, The Netherlands; 3 Department of Microbiology, Faculty of Medicine, Khon Kaen University, Khon Kaen, Thailand

**Keywords:** Dual species, Biofilm, Dental caries, Periodontitis, Phytosphingosine, Diseases, Microbiology

## Abstract

Oral biofilms are major etiological factors in dental caries and periodontitis and are often resistant to conventional anti-microbial therapies, particularly in mixed-species communities. Phytosphingosine (PHS), a naturally occurring bioactive sphingolipid, has demonstrated anti-microbial activity against several pathogens; however, its effects on mixed-species oral biofilms and inflammatory responses remain unclear. This study investigated the in vitro anti-microbial, anti-biofilm, and immunomodulatory activities of PHS against *Streptococcus mutans* and *Aggregatibacter actinomycetemcomitans* in single- and dual-species biofilms. Confocal laser scanning microscopy revealed a significant reduction in biofilm matrix formation in both single- and dual-species biofilms treated with PHS compared with untreated controls (*P* < 0.001). Cytotoxicity analysis demonstrated that cell viability remained above 90% following exposure to 5 µg/ml PHS for 24 h. Furthermore, PHS pretreatment significantly attenuated LPS-induced IL-6 and TNF-α production (*P* < 0.001). Collectively, these findings suggest that PHS is a promising dual-function agent with anti-microbial and anti-inflammatory properties and may have potential as an adjunctive therapeutic strategy for biofilm-associated oral diseases.

**Supplementary Information:**

The online version contains supplementary material available at 10.1038/s41598-026-55714-3.

## Introduction

Oral biofilms are complex, structured microbial communities that play a central role in the pathogenesis of prevalent chronic oral diseases, including dental caries and periodontitis^1^. The WHO global oral health status report (2022) estimates that nearly 3.5 billion people worldwide are affected by oral diseases, with dental caries and periodontal disease being particularly prevalent^2^.

*Streptococcus mutans* is a primary etiological agent of dental caries due to its strong acidogenic capacity and robust production of extracellular polymeric substances (EPS)^3^. In contrast, *Aggregatibacter actinomycetemcomitans* is closely associated with aggressive periodontitis (AgP) characterized by dysregulation of host immune responses and rapid periodontal tissue destruction^4^, which predominantly affects adolescents and young adults compared with other types of periodontal disease^5^. *A. actinomycetemcomitans* primarily colonizes the oral mucosa, tooth surfaces, and subgingival sites^6^, whereas *S. mutans* is mainly associated with supragingival biofilms^7^. However, bacteria from these niches are continuously dispersed into saliva, with subgingival species further disseminated via gingival crevicular fluid^8^, thereby increasing the likelihood of co-aggregation and contributing to disease progression. This is consistent with their clinically observed co-occurrence in the saliva of patients with AgP and proximal caries^9^. Increasing evidence suggests that the coexistence of these microorganisms within mixed-species biofilms may enhance biofilm stability and pathogenicity, thereby complicating disease control^10,11^. Dental plaque in dental caries and periodontitis has been well documented, particularly in relation to root caries and periodontal pockets^10^. Co-culture studies have demonstrated interspecies communication, in which *A. actinomycetemcomitans* can modulate quorum-sensing pathways of *S. mutans*, leading to enhanced biofilm formation^11^. Such interactions may influence the balance between oral health and dysbiosis^12^. However, despite a reported positive correlation between periodontal disease and dental caries, patients with AgP have been shown to exhibit reduced *S. mutans* abundance^10,13^. Although an association between AgP and dental caries has been reported, the precise interactions, such as protein-protein communication between *A. actinomycetemcomitans* and *S. mutans* within mixed-species biofilms, as well as their contributions to biofilm structural integrity, remain incompletely understood.

Conventional anti-microbial therapies, including antibiotics and antiseptics such as chlorhexidine (CHX), are commonly used to manage biofilm-associated oral infections^14^. However, their effectiveness is often limited by poor penetration into the biofilm matrix, the presence of metabolically dormant persister cells, and undesirable cytotoxic effects on host tissues^15,16^. Moreover, excessive use of antibiotics contributes to the emergence of anti-microbial resistance^17^, highlighting the urgent need for alternative therapeutic strategies that can effectively target biofilms while minimizing cytotoxicity.

Phytosphingosine (PHS) is a naturally occurring bioactive sphingolipid found in mammalian tissues, yeast and plants and has attracted attention due to its anti-microbial defence and anti-inflammatory properties^18^. Previous studies have demonstrated its anti-microbial efficacy against *S. mutans*^19^, *Candida albicans*^20^, and various other pathogenic bacteria^21^. Additionally, the anti-adherence capacity of PHS on titanium surfaces used for dental implants has recently been reported^22^. The anti-microbial mechanism of PHS involves its interaction with negatively charged phospholipids in microbial cell membranes, leading to membrane destabilization, disruption of intracellular components, and ultimately cell death^23,24^. Despite these promising findings, the effects of PHS on mixed-species oral biofilms have not been fully investigated. In addition, the potential of PHS to modulate host inflammatory responses associated with periodontal disease remains largely unexplored.

To our knowledge, this study is among the first to evaluate the dual antimicrobial and immunomodulatory effects of PHS in a cross-niche dual-species oral biofilm model. Accordingly, this study aimed to investigate the anti-microbial and anti-biofilm activities of PHS against *S. mutans* and *A. actinomycetemcomitans* in both single- and dual-species in vitro models. Comparative proteomic analysis was performed to investigate interspecies interactions within dual-species biofilms. The immunomodulatory effects of PHS were assessed by quantifying interleukin-6 (IL-6) and tumor necrosis factor-alpha (TNF-α) levels in LPS-stimulated human gingival fibroblasts. Biofilm matrix disruption was evaluated by confocal laser scanning microscopy. The proteomic results suggest that *A. actinomycetemcomitans* may suppress the growth of *S. mutans* in dual-species biofilms by downregulating pathways involved in nutrient sensing and metabolic homeostasis, providing novel insights into competitive interactions between cariogenic and periodontal pathogens. PHS exhibited potent anti-microbial activity against planktonic cells and effectively disrupted the biofilm matrix in both single- and dual-species cultures, while demonstrating minimal cytotoxicity toward human gingival fibroblasts. Furthermore, PHS pretreatment significantly reduced IL-6 and TNF-α expression. Collectively, these findings highlight the dual anti-microbial and host-modulatory properties of PHS, supporting its potential as a multifunctional therapeutic agent for biofilm-associated oral diseases.

## Results

### Antimicrobial susceptibility testing

The minimum inhibitory concentration (MIC) and minimum bactericidal concentration (MBC) were determined to assess the susceptibility of all tested bacteria in planktonic cultures (Table 1). Doxycycline (DOX) exhibited low MIC values against *S. mutans*, *A. actinomycetemcomitans*, and dual-species cultures. However, in presence of *S. mutans*, the MBC of DOX increased to 64 µg/ml. Amoxicillin (AMX) also exhibited low MIC values against all tested conditions; nevertheless, the MBC of AMX was consistently 64 µg/ml across all cultures. In contrast, PHS showed greater anti-bacterial activity against *S. mutans* than against *A. actinomycetemcomitans.* Notably, the MBC of PHS against *S. mutans* was at least 64-fold lower than those of the antibiotics tested. Moreover, PHS exhibited bactericidal activity against both *A. actinomycetemcomitans* and dual-species cultures at a concentration of 4 µg/ml. No MIC or MBC values were observed for the vehicle control. These findings suggest that, under the conditions tested, PHS demonstrated stronger bactericidal activity than DOX and AMX, as indicated by MBC values.

**Table 1:** Susceptibility testing of AMX, DOX and PHS against planktonic cultures. N/A: Not applicable.

Bacterial strains	AMX (µg/ml)		DOX (µg/ml)		PHS (µg/ml)		Vehicle control	
	MIC	MBC	MIC	MBC	MIC	MBC	MIC	MBC
*S. mutans*	0.25	64	0.125	64	0.5	1	N/A	N/A
*A. actinomycetemcomitans*	1	64	0.25	16	2	4	N/A	N/A
Dual culture	1	64	0.25	64	2	4	N/A	N/A

### Time-kill kinetics of PHS against planktonic cultures

**Fig. 1:**
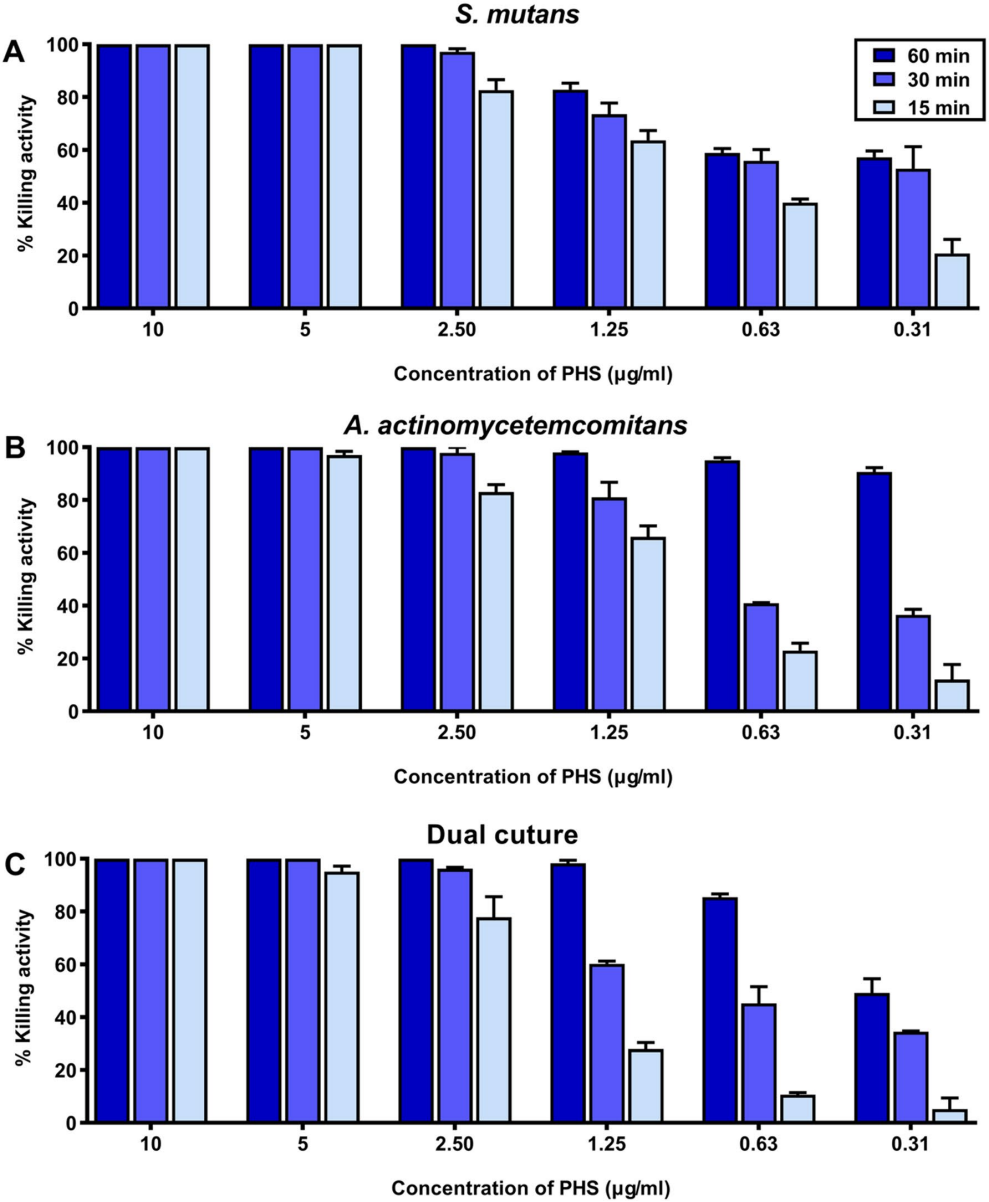
Killing activity of PHS against planktonic cultures. Bacterial cells of (**A**) *S. mutans*, (**B**) *A. actinomycetemcomitans*, and (**C**) dual-species cultures were incubated with PHS at concentrations ranging from 0.31 to 10 µg/ml for 15, 30, and 60 min. Data are presented as mean ± SD of three independent experiments, each performed in duplicate.

The bactericidal activity of PHS against single- and dual-species cultures was evaluated (Fig. 1). PHS exhibited clear time- and concentration-dependent killing against all tested planktonic cultures. More than 90% bacterial killing was observed following exposure to PHS at 2.5 µg/ml for 30 min. At 5 µg/ml, PHS achieved killing rates of 95.1 ± 2.1%, 97.1 ± 1.4%, and 100% within 15 min against dual-species cultures, *A. actinomycetemcomitans*, and *S. mutans*, respectively. Moreover, treatment with PHS at 10 µg/ml resulted in complete bacterial killing under all tested conditions.

### Biofilm-forming capacity

**Fig. 2:**
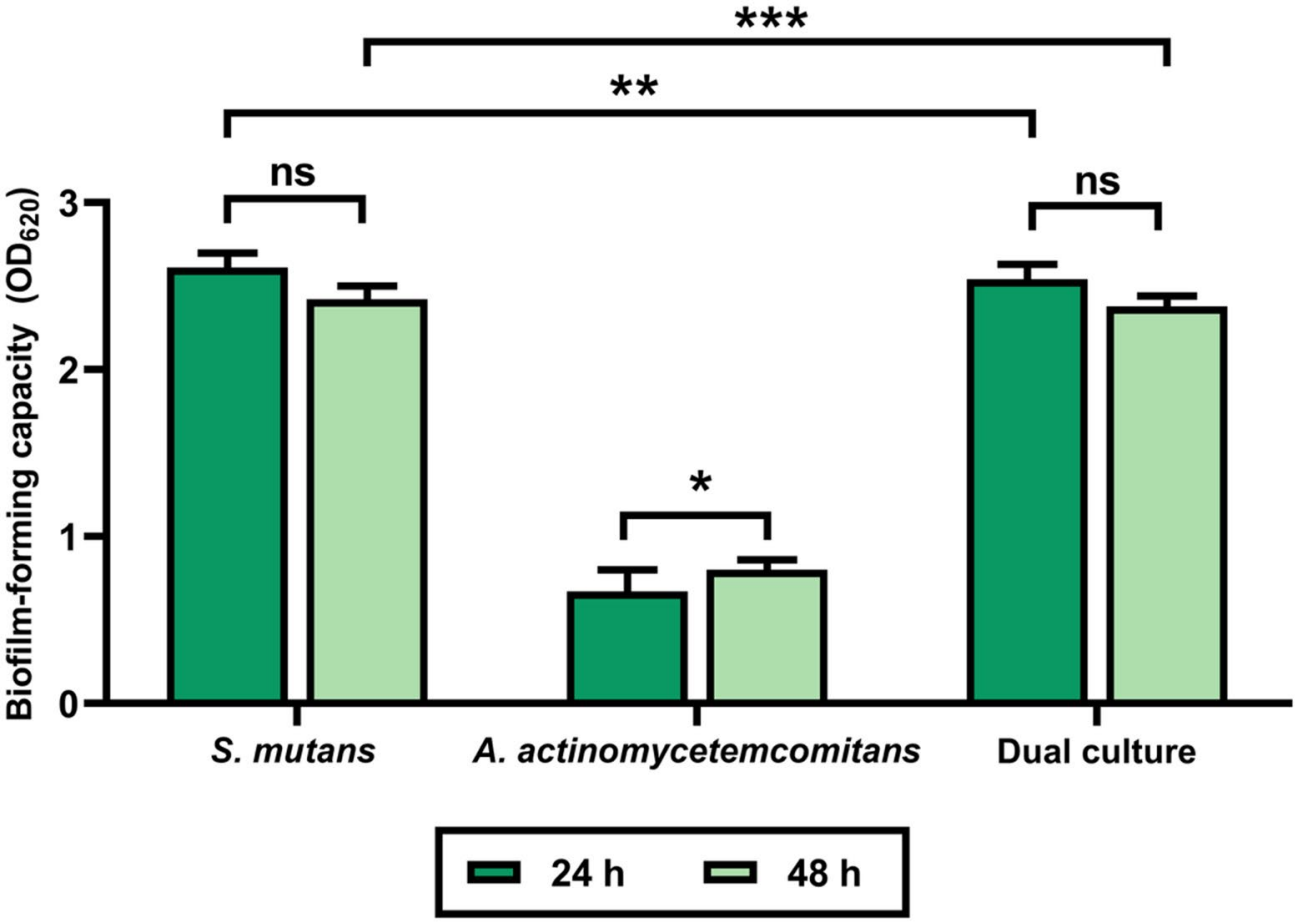
Quantification of biofilm formation. Total biofilm biomass of single-species and dual-species cultures at 24 and 48 h was quantified using the crystal violet assay. Data are presented as mean ± SD from three independent experiments, each performed in 8 replicates. ns = not significant, *P < 0.05, **P < 0.01 and *** *P* < 0.001.

The biofilm-forming capacity of single- and dual-species cultures was evaluated using the crystal violet staining assay (Fig. 2). At 24 h, the highest level of biofilm formation was observed in *S. mutans* (OD₆₂₀, mean ± SD: 2.61 ± 0.09), whereas substantially lower biofilm formation was detected in *A. actinomycetemcomitans* (0.67 ± 0.13). Biofilm formation in dual-species cultures at 24 h (2.54 ± 0.09) was significantly lower than that observed in *S. mutans* single-cultures (*P* < 0.01). At 48 h, a slight reduction in biofilm biomass was observed in *S. mutans* alone (2.42 ± 0.08) and in dual-species biofilms (2.38 ± 0.06); although these differences were not statistically significant. In contrast, biofilm formation by *A. actinomycetemcomitans* was significantly increased to 0.80 ± 0.06 at 48 h (*P* < 0.05). Compared with *S. mutans* at 48 h, the dual-species biofilm remained significantly lower (*P* < 0.001), consistent with the reduction observed at 24 h. These results suggest that co-culture with *A. actinomycetemcomitans* partially reduced *S. mutans* biofilm formation.

### Morphology of dual-species biofilms

**Fig. 3:**
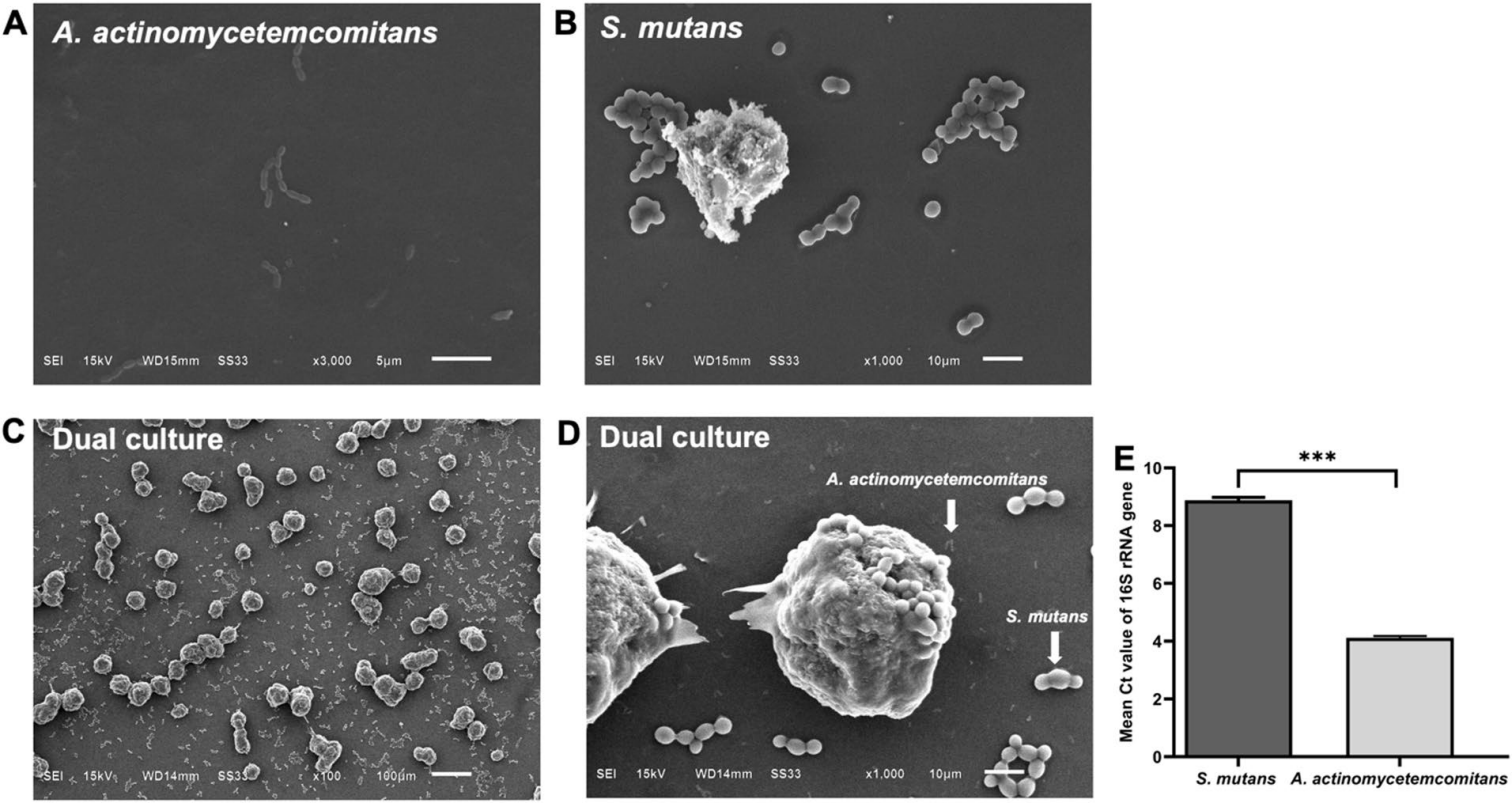
The morphology of single- and dual-species biofilms at 24 h and bacterial composition in dual-species biofilms. Biofilms of (**A**) *A. actinomycetemcomitans* at 3000**×** magnification, (**B**) *S. mutans* at 1000**×** magnification, (**C**) dual-species at 100**×** magnification, and (**D**) dual-species at 1000**×** magnification were visualized by SEM. (**E**) The relative abundance of each bacterial species within dual-species biofilms was quantified by qRT-PCR. Data are presented as mean ± SD of three independent experiments, each performed in duplicate. ***P < 0.001.

Scanning electron microscopy (SEM) was used to examine the structural characteristics of single- and dual-species biofilms at 24 h (Fig. 3). In single-species biofilms, *A. actinomycetemcomitans* observed at 3000× magnification (Fig. 3A) appeared as small, sparsely distributed cells with limited surface coverage. In contrast, the *S. mutans* biofilms imaged at 1000× magnification (Fig. 3B) exhibited dense bacterial aggregates forming well-defined microcolonies, with scattered individual cells surrounding these structures.

In dual-species biofilms, images captured at 100× and 1,000× magnification (Fig. 3C and D) revealed a biofilm architecture predominantly dominated by *S. mutans*. Large, densely clustered microcolonies formed prominent “cap-and-stalk”–like structures that accounted for the majority of the biofilm biomass. Regions of lower cell density associated with *S. mutans* were interspersed between these clusters. In contrast, smaller *A. actinomycetemcomitans* cells were detected only sporadically on the surface and appeared to contribute minimally to the overall biofilm architecture, primarily localizing around the *S. mutans*-dominated structures.

### Bacterial proportion

The bacterial composition of dual-species biofilms was analyzed by qRT-PCR targeting species-specific 16S rRNA gene (Fig. 3E). In dual-species biofilms, *A. actinomycetemcomitans* (Ct = 4.12 ± 0.06) exhibited significantly lower Ct values than *S. mutans* (8.80 ± 0.10) (*P* < 0.001), indicating a higher relative abundance of *A. actinomycetemcomitans* within the biofilm. These findings suggest that the relative abundance of *S. mutans* is reduced under co-culture conditions.

### Proteomic profiling of single- and dual-species biofilms

**Fig. 4:**
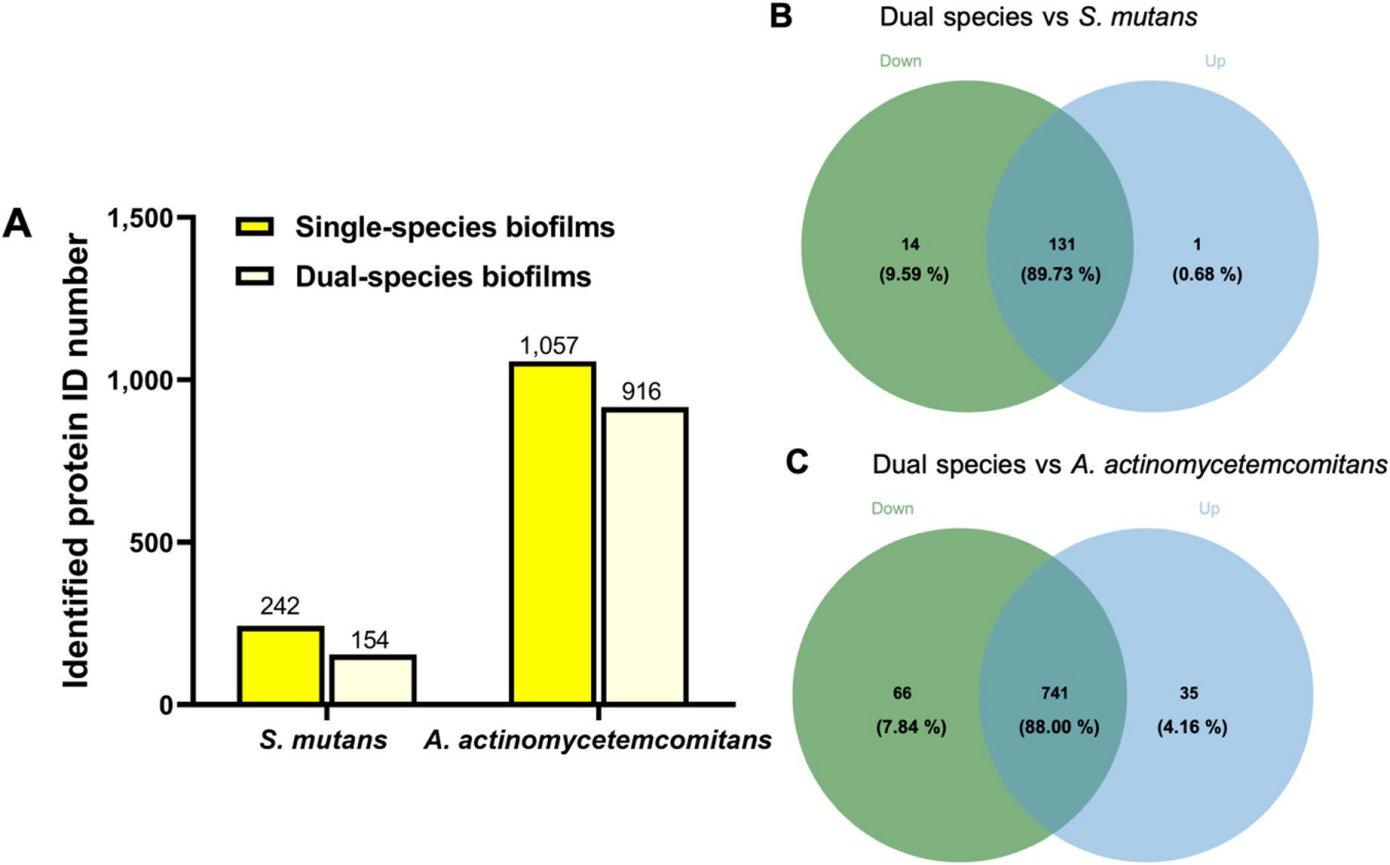
Comparative protein expression profiles of single-species and dual-species biofilms. (**A**) Total numbers of proteins identified in *A. actinomycetemcomitans* and *S. mutans* under single-species and dual-species biofilm conditions. Proteins displaying an absolute log₂ fold change (log₂FC) ≥ 1.2 were defined as differentially expressed. Venn diagram analyses depict the distribution of up- and down-regulated proteins in dual-species biofilms relative to (**B**) *S. mutans* and (**C**) *A. actinomycetemcomitans* single-species biofilms.

To characterize the differential protein expression in *A. actinomycetemcomitans* and *S. mutans*, proteomic analysis was performed on single- and dual-species biofilms (Fig. 4). In single-species biofilms, a total of 1,057 proteins were identified in *A. actinomycetemcomitans* and 242 proteins in *S. mutans* (Fig. 4A). In dual-species biofilms, the number of detected proteins decreased to 916 for *A. actinomycetemcomitans* and 154 for *S. mutans*. These results indicate a reduction in detectable protein expression in both species under co-culture conditions, with a more pronounced decrease observed in *S. mutans*.

Differential protein expression was determined by comparing protein abundance between single- and dual-species biofilms (Supplementary Table S1). Proteins exhibiting an absolute log₂ fold change (log₂FC) ≥ 1.2 were classified as up- or down-regulated. In *S. mutans*, comparison between biofilm conditions revealed one up-regulated and fourteen down-regulated proteins in the dual-species biofilm (Fig. 4B). The downregulated proteins (Table 2) were mainly associated with protein synthesis (ribosomal proteins *rpsP* and *rplD*), transcriptional and translational regulation (*sigA*, *tsf*, *codY*), membrane transport and nutrient uptake (ABC transporters), and central metabolism (glycerol dehydrogenase). These changes suggest a reduction in metabolic activity and growth of *S. mutans* under dual-species conditions, potentially due to nutrient competition or antagonistic interactions with *A. actinomycetemcomitans*. In contrast, the upregulation of glucosyltransferase (*gtfC*), a key enzyme involved in extracellular polysaccharide (EPS) synthesis, may indicate a compensatory response to maintain biofilm structure under stress.

**Table 2:** The majority of differentially expressed proteins in dual-species biofilms compared with *S. mutans* single-species biofilms.

Protein accession	Description	Gene	Fold change
Up-regulated proteins			
P13470	Glucosyltransferase-SI	*gtfC*	1.44
Down-regulated proteins			
Q8DW31	ABC transporter, membrane protein	SMU_248	− 2.86
Q8DT14	67 kDa myosin-crossreactive streptococcal antigen-like protein	SMU_1584c	− 2.12
Q8DVJ3	Glycerol dehydrogenase	*gldA*	− 2.04
Q8DUN9	Small ribosomal subunit protein bS16	*rpsP*	− 1.50
Q8CWZ6	Trigger factor	*tig*	− 1.50
Q8DT33	Trk system potassium uptake protein TrkA	*trk*	− 1.50
Q8DS17	Large ribosomal subunit protein uL4	*rplD*	− 1.49
O33662	RNA polymerase sigma factor SigA	*sigA*	− 1.40
P59388	Global transcriptional regulator CodY	*codY*	− 1.35
Q8DS12	Elongation factor Ts	*tsf*	− 1.34

In *A. actinomycetemcomitans*, comparative analysis demonstrated that 35 proteins were up-regulated and 66 proteins were down-regulated in dual-species biofilms relative to single-species biofilms (Fig. 4C). A strong enrichment of proteins involved in central metabolism and biosynthesis was observed (Table 3), including those related to glycolysis (*gap*, *pgi*), amino acid metabolism (*glyA*, *thrC*), nucleotide synthesis (*nrdB*), and energy metabolism (phosphate acetyltransferase). In contrast, proteins associated with protein synthesis (ribosomal proteins), transport systems (ABC transporters), and certain metabolic pathways were downregulated. Together, these patterns suggest a shift toward a metabolically efficient, adaptive state, which may support the persistence of *A. actinomycetemcomitans* and its competitive advantage over *S. mutans *in the dual-species environment.

**Table 3:** The majority of differentially expressed proteins in dual-species biofilms compared with *A. actinomycetemcomitans* single-species biofilms.

Protein accession	Description	Gene	Fold change
Up-regulated proteins			
P34894	Serine hydroxymethyltransferase	*glyA*	6.60
A0A5D0EJY8	Glyceraldehyde-3-phosphate dehydrogenase	*gap*	5.01
A0A142G2D5	Carboxy terminal-processing peptidase	ACT75_09940	4.67
A0A5D0EL46	Ribonucleoside-diphosphate reductase	*nrdB*	3.37
A0A5D0EL39	Glucose-6-phosphate isomerase	*pgi*	3.06
A0A142FYR0	Threonine synthase	*thrC*	2.49
A0A5D0EKN6	Phosphate acetyltransferase	ACT75_01260	2.29
A0A5D0ENA9	NrfG protein	ACT75_08245	2.25
A0A5D0EK21	Multiphosphoryl transfer protein	ACT75_08325	2.20
A0A142FXI8	DNA-binding protein	ACT75_00540	1.95
Down-regulated proteins			
A0A5D0EKR4	Ribosomal protein uS12 methylthiotransferase RimO	*rimO*	− 5.35
A0A142G0C4	Amino acid ABC transporter permease	ACT75_05945	− 3.87
A0A5D0EJE0	ADP-L-glycero-D-manno-heptose-6-epimerase	*rfaD*	− 3.24
A0A142FZ42	Small ribosomal subunit protein bS20	*rpsT*	− 3.24
P67897	Small ribosomal subunit protein uS19	*rpsS*	− 3.07
A0A142FYR2	Protein HflK	*hflK*	− 2.93
A0A142FZ95	Large-conductance mechanosensitive channel	*mscL*	− 2.76
A0A142FYT0	Formate acetyltransferase	*pflB*	− 2.66
A0A142FXU0	DUF4198 domain-containing protein	ACT75_01070	− 2.60
A0A142FXL2	Integration host factor subunit beta	*ihfB*	− 2.54

**Fig. 5:**
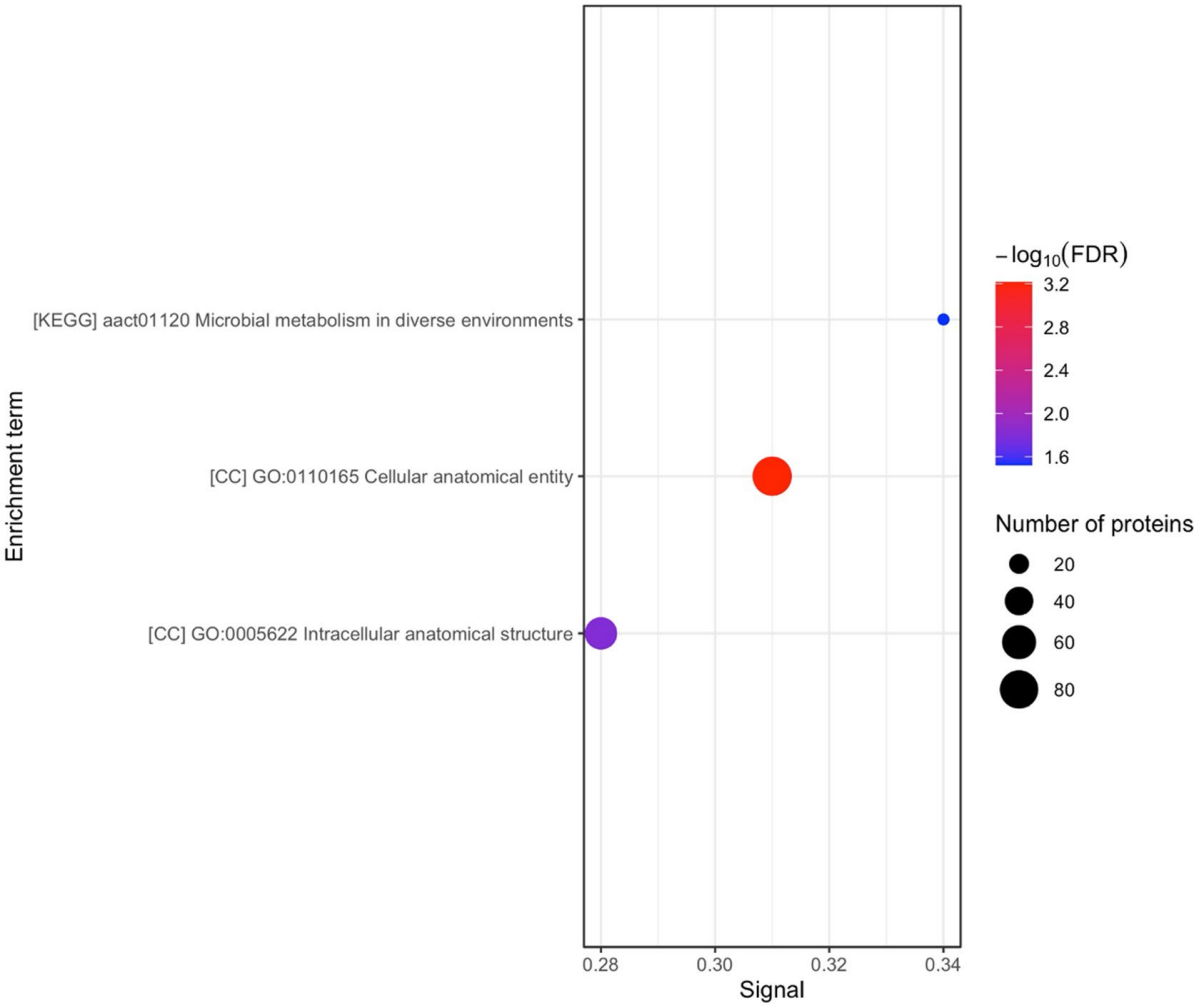
Functional enrichment analysis of differentially expressed proteins in dual-species biofilms relative to *A. actinomycetemcomitans* single-species biofilms. Enrichment analysis was conducted using the STRING database (version 12.0), with protein accession numbers mapped to *S. mutans* UA159 or *A. actinomycetemcomitans*. Enriched Gene Ontology terms and KEGG pathways were considered statistically significant at a false discovery rate (FDR) < 0.05.

Functional enrichment analysis using STRING was performed to classify the differentially expressed proteins according to Gene Ontology (GO) terms and KEGG pathways, providing insight into their biological roles (Fig. 5). Significant enrichment was observed exclusively among the up- and down-regulated proteins of *A. actinomycetemcomitans* in dual-species biofilms relative to single-species biofilms. The enriched GO terms were predominantly associated with cellular component categories, including cellular anatomical entity (GO:0110165) and intracellular anatomical structure (GO:0005622). In addition, significant enrichment was identified in the KEGG pathway related to microbial metabolism in diverse environments (aact01120). These findings indicate that metabolic adaptation may play a critical role in supporting the growth and persistence of *A. actinomycetemcomitans* within dual-species biofilms.

### Anti-biofilm formation

**Fig. 6:**
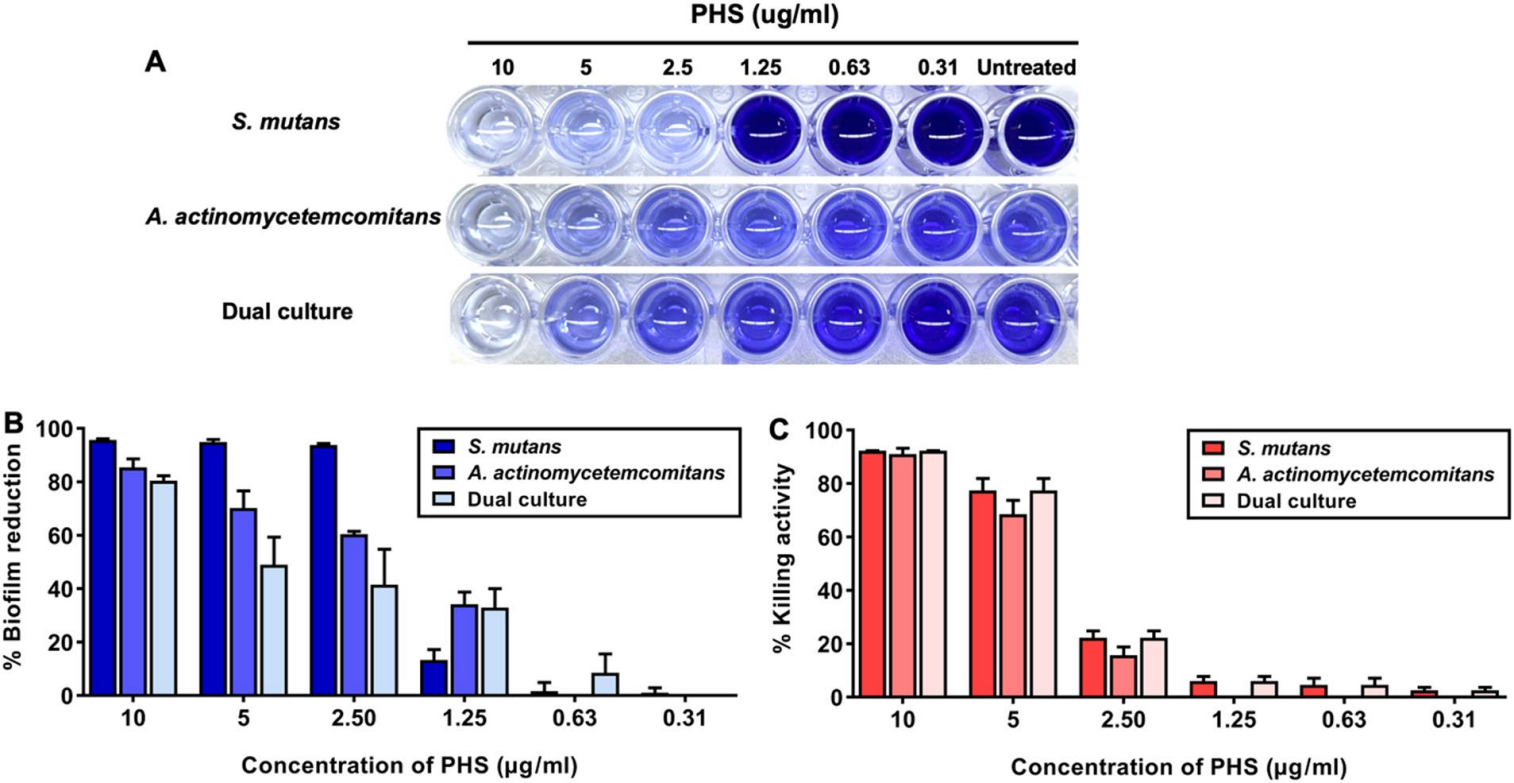
Anti-biofilm activity of PHS against single- and dual-species biofilms. (**A**) Bacterial cultures were exposed to PHS (0.31–10 µg/ml) at 37 °C under 5% CO₂ for 24 h, followed by quantification of total biofilm biomass using the crystal violet assay. (**B**) Biofilm biomass reduction was calculated by comparing PHS-treated groups with untreated controls. (**C**) The bactericidal activity of PHS against biofilm-embedded cells was evaluated using the MTT assay. Data are presented as mean ± SD of three independent experiments, each performed in duplicate.

The inhibitory effects of PHS on biofilm formation are shown in Fig. 6. After 24 h of incubation, crystal violet staining demonstrated a clear concentration-dependent reduction in biofilm biomass across all tested conditions (Fig. 6A). Among the tested species, *S. mutans* exhibited the greatest sensitivity to PHS treatment. At a concentration of 10 µg/ml, PHS reduced biofilm biomass by 95.7 ± 0.4% for *S. mutans*, 85.4 ± 3.2% for *A. actinomycetemcomitans*, and 80.4 ± 1.9% for dual-species biofilms (Fig. 6B). The effect of PHS on biofilm cell viability was assessed using the MTT assay. At 10 µg/ml, PHS achieved killing efficiencies of 93.2 ± 0.3% for *S. mutans*, 91.0 ± 2.2% for *A. actinomycetemcomitans*, and 92.2 ± 0.1% for dual-species biofilms (Fig. 6C). Notably, treatment with 5 µg/ml PHS resulted in > 70% reduction in metabolic activity across all tested biofilm conditions. These results indicate that PHS effectively suppresses biofilm formation and reduces the viability of biofilm-embedded cells.

### Inhibition of bacterial attachment

**Fig. 7:**
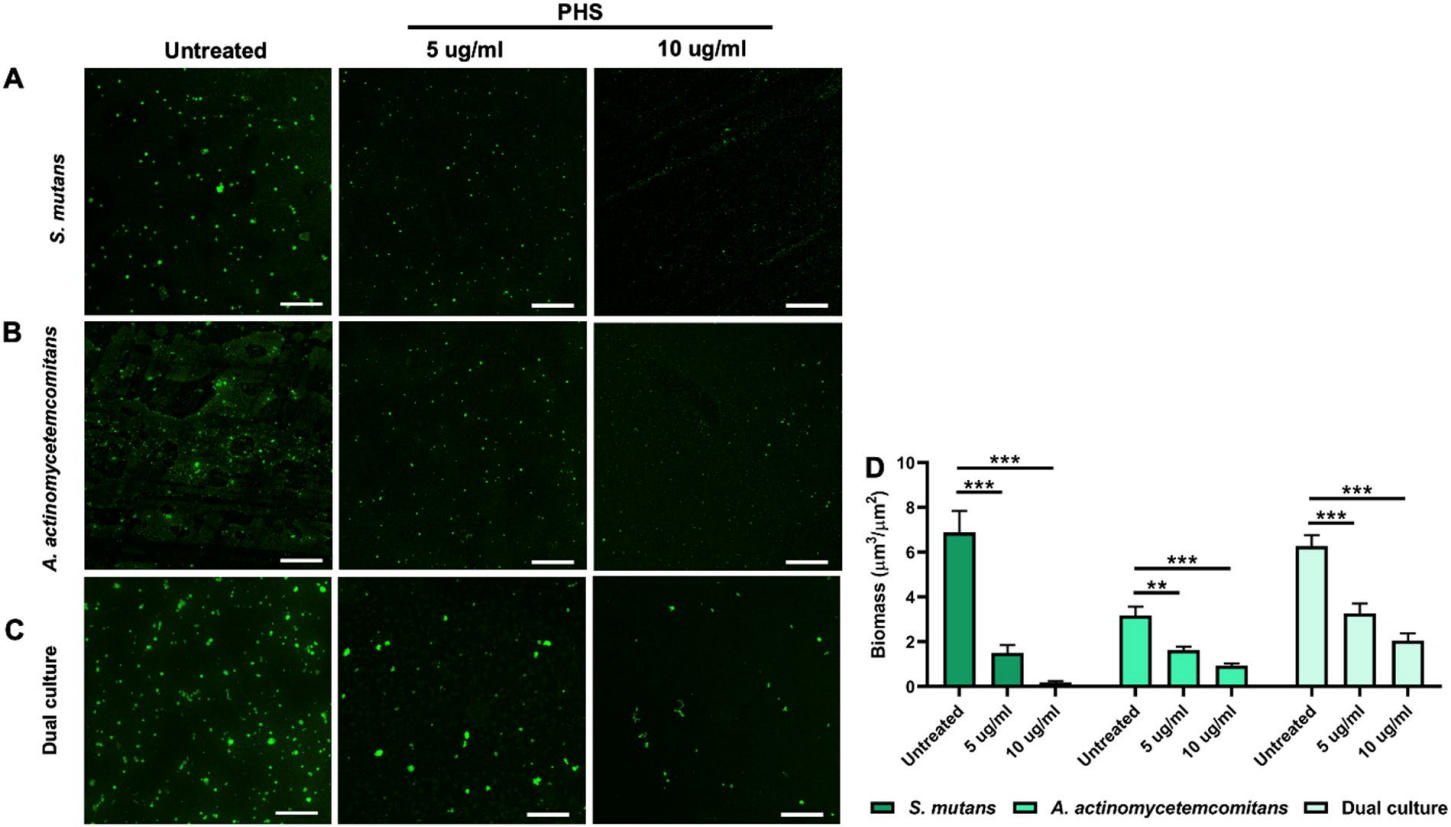
Effect of PHS on the bacterial attachment in single- and dual-species biofilms. Bacterial cultures of (**A**) *S. mutans*, (**B**) *A. actinomycetemcomitans,* and (**C**) dual-species culture were exposed to PHS (5 and 10 µg/ml) at 37 °C under 5% CO₂ for 3 h. Viable attached bacterial cells were visualized by staining with SYTO™ Green fluorescent nucleic acid stain. (**D**) The biomass of green fluorescence, representing viable attached cells, was quantified using COMSTAT analysis. Data are presented as mean ± SD of three independent experiments, each performed in duplicate. **P < 0.01 and ***P < 0.001. Scale bar indicates 100 μm.

The effect of PHS on bacterial attachment was evaluated under both single- and dual-species conditions (Fig. 7). In untreated controls, strong green fluorescence was observed, indicating extensive attachment of viable bacterial cells. In contrast, PHS treatment resulted in a clear, dose-dependent reduction in bacterial attachment across all conditions. In single-species cultures of *S. mutans*, untreated samples showed dense and uniform surface attachment (Fig. 7A). Treatment with 5 µg/ml PHS markedly reduced the number of adherent cells (*P* < 0.001), while treatment with 10 µg/ml resulted in only sparse residual attachment (*P* < 0.001). A similar pattern of attachment inhibition was observed in *A. actinomycetemcomitans* single-species cultures (Fig. 7B). In the dual-species condition, PHS treatment also significantly reduced the total attached biomass at both 5 and 10 µg/ml (*P* < 0.001) (Fig. 7C). These results demonstrate that PHS effectively inhibits early bacterial attachment in both single- and dual-species systems.

### Biofilm matrix reduction

**Fig. 8:**
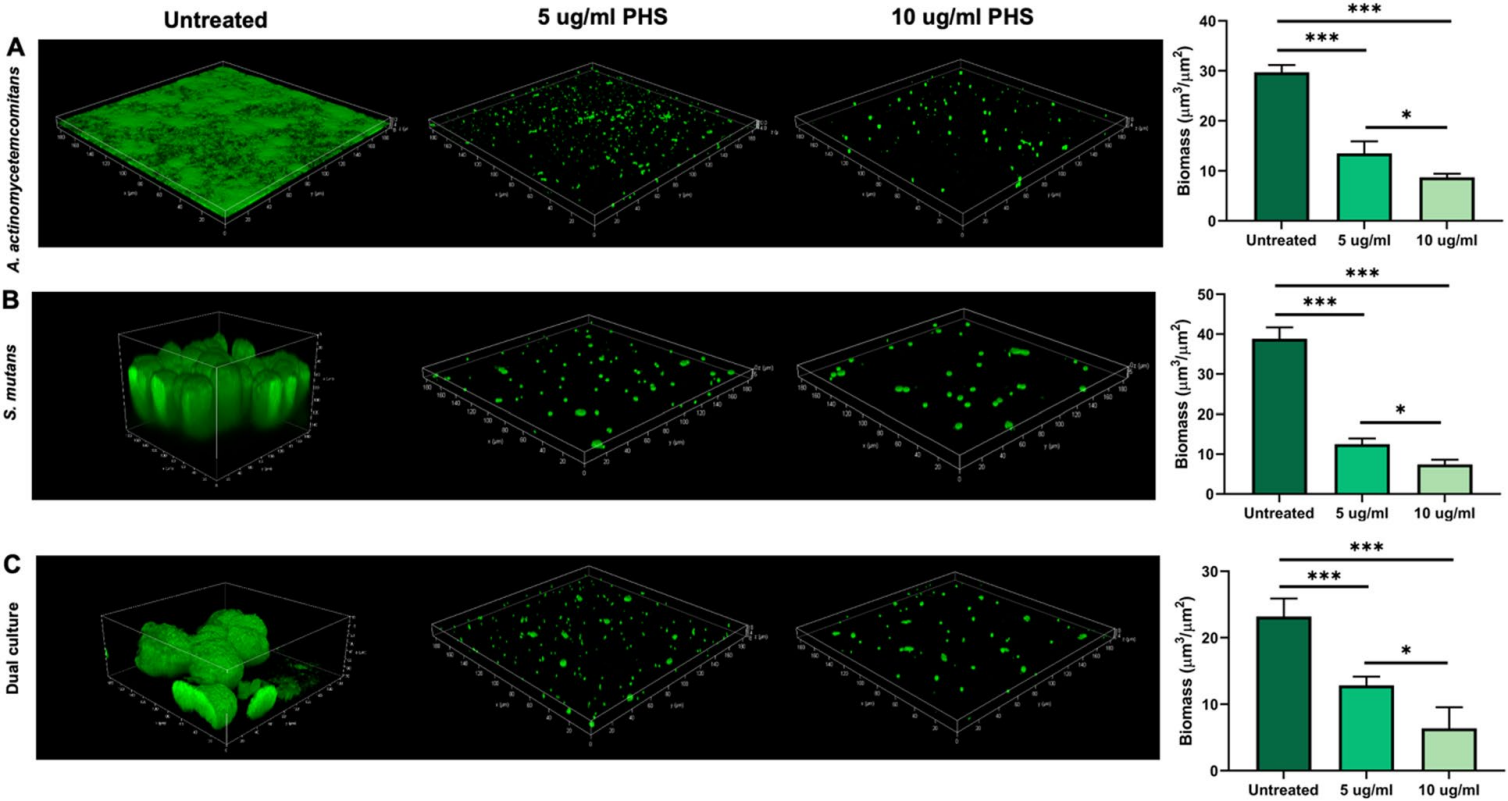
Effect of PHS on the biofilm matrix in single- and dual-species biofilms. Bacterial cultures of (**A**) *A. actinomycetemcomitans*, (**B**) *S. mutans,* and (**C**) dual-species culture were treated with PHS (5 and 10 µg/ml) and incubated at 37 °C under 5% CO₂ for 24 h. Biofilm matrix components were stained with FITC–ConA and visualized using CLSM. Quantitative analysis of biofilm biomass was performed using COMSTAT. Data are presented as mean ± SD of three independent experiments, each performed in duplicate. *P < 0.05 and *** *P* < 0.001.

The FITC–ConA green fluorescence was used to visualize the extracellular matrix of single- and dual-species biofilms following exposure to PHS (Fig. 8). In single-species cultures, untreated *A. actinomycetemcomitans* biofilm exhibited a flat and relatively smooth architecture (Fig. 8A). Treatment with PHS at 5 and 10 µg/ml significantly reduced the biofilm matrix compared with untreated controls (*P* < 0.001). Similarly, PHS markedly disrupted the thicker, tower-like structures formed by *S. mutans* biofilms (Fig. 8B) (*P* < 0.001). In dual-species biofilms, PHS treatment also induced a pronounced, dose-dependent decrease in biofilm matrix (Fig. 8C) (*P* < 0.001). These findings indicate that PHS effectively disrupts both flat and complex biofilm architectures in single- and dual-species biofilms.

### Cellular cytotoxicity of PHS

**Fig. 9:**
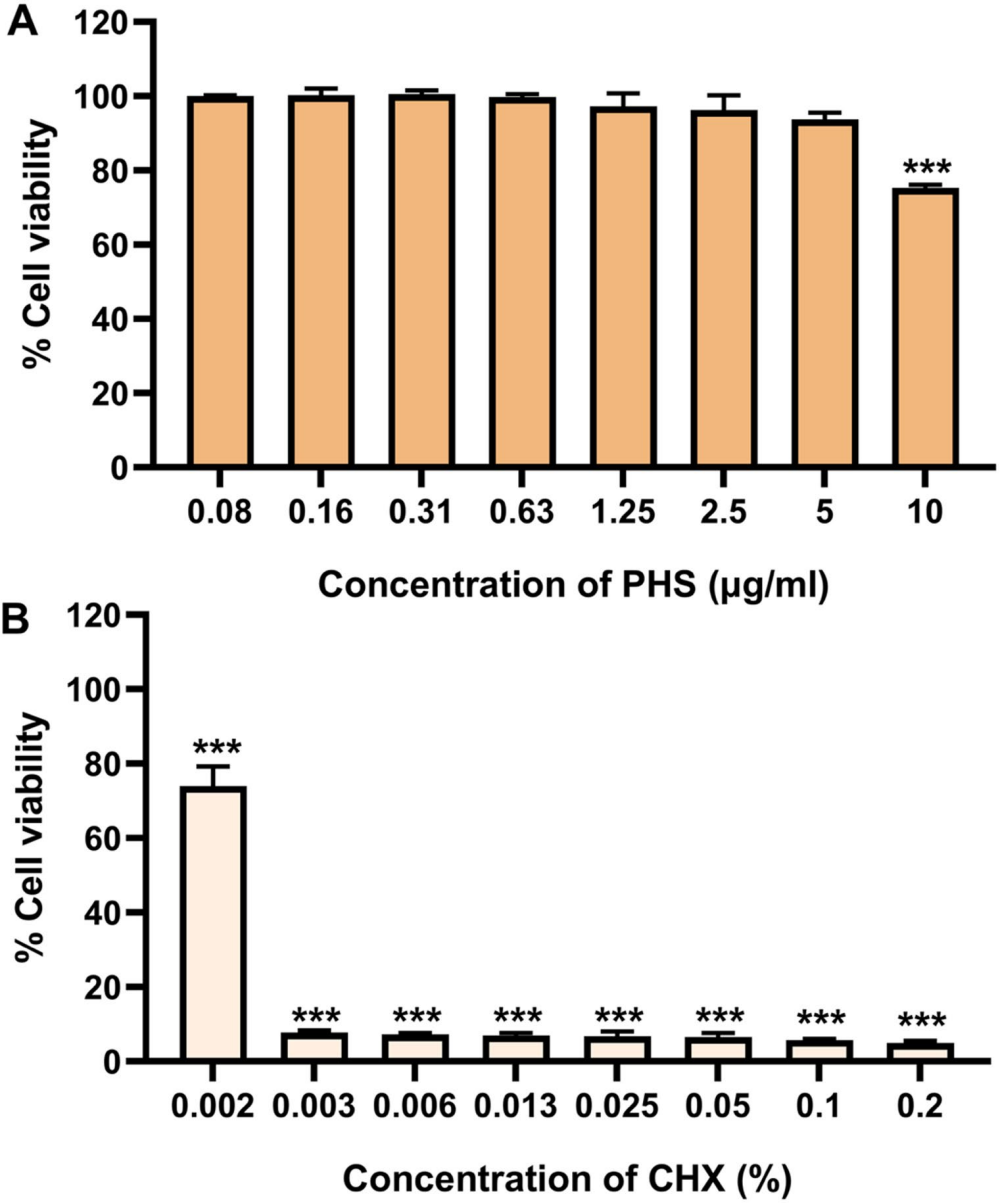
Cytotoxicity of PHS on HGF-1 cells. Cells were incubated with (**A**) PHS (0.08–10 µg/ml) and (**B**) CHX (0.002%-0.2%) for 24 h. The cell viability was assessed using the MTT assay. Data are presented as mean ± SD of three independent experiments, each performed in duplicate. ***P < 0.001.

Human gingival fibroblasts-1 (HGF-1) were used to evaluate the cytotoxic effects of PHS (Fig. 9). After 24 h of exposure, treatment with 10 µg/ml PHS resulted in a cell viability of 75.2 ± 0.9%, while viability remained high (93.7 ± 1.8%) at 5 µg/ml. In contrast, 0.2% CHX markedly reduced cell viability to 4.91 ± 0.66%. Thus, 5 µg/ml PHS was selected for subsequent experiments due to its minimal cytotoxicity and its effective anti-biofilm activity.

### Anti-inflammatory effects of PHS on LPS-induced cytokine production

**Fig. 10:**
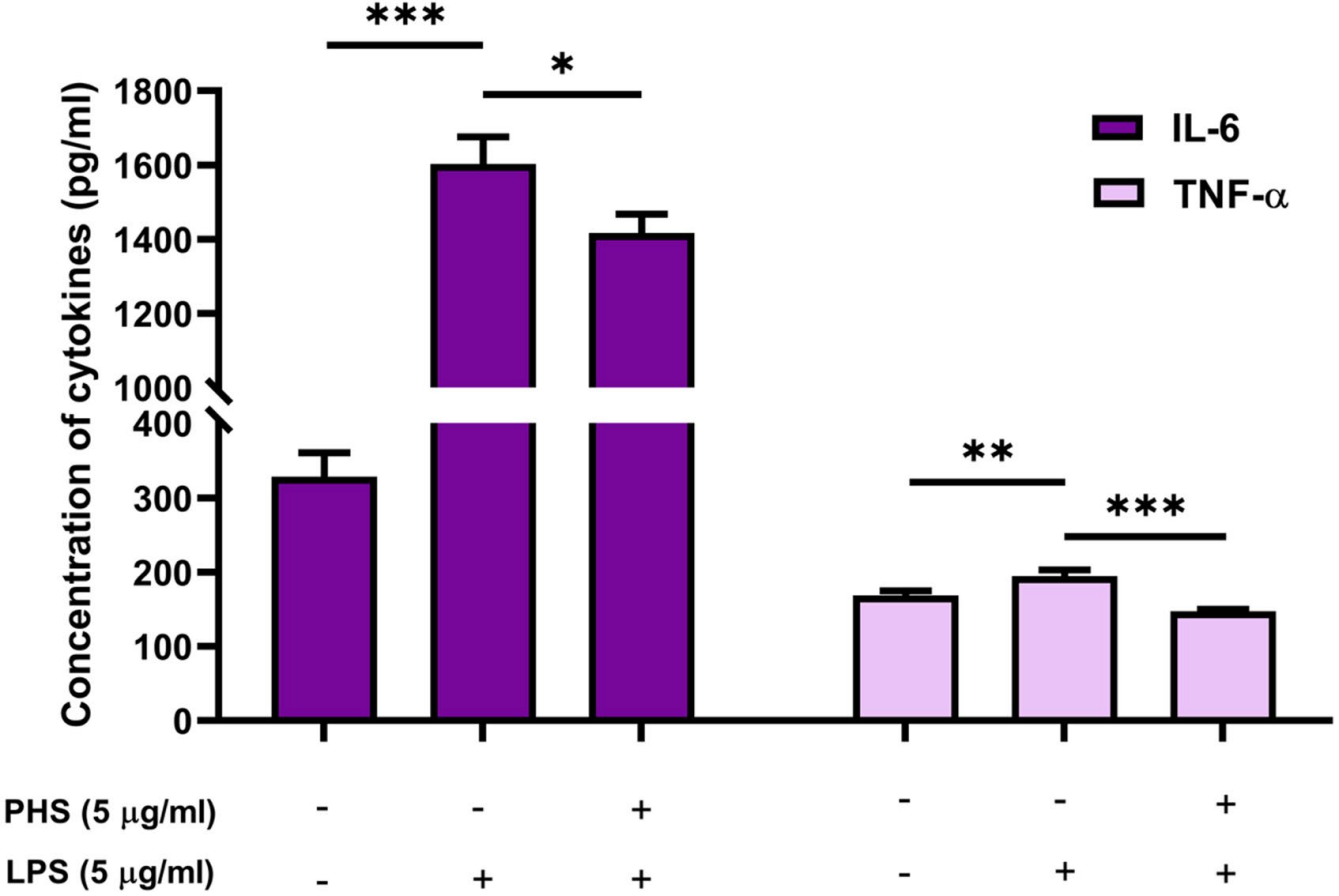
Attenuation of pro-inflammatory cytokine production by PHS in LPS-induced cells. HGF-1 cells were pretreated with PHS (5 µg/ml) for 1 h prior to stimulation with LPS (5 µg/ml). The concentrations of pro-inflammatory cytokines (IL-6 and TNF-α) were quantified using ELISA. Data are presented as mean ± SD from three independent experiments, each performed in duplicate. *P < 0.05, **P < 0.01, and ***P < 0.001.

The in vitro anti-inflammatory activity of PHS against LPS-induced inflammation was evaluated using ELISA (Fig. 10). LPS stimulation of HGF-1 cells resulted in significantly increased secretion of IL-6 (*P* < 0.001) and TNF-α (*P* < 0.01) compared with the uninduced control. In contrast, pretreatment of HGF-1 cells with 5 µg/ml PHS for 1 h prior to LPS exposure significantly reduced IL-6 production (*P* < 0.05) relative to the LPS-treated group. A marked reduction in TNF-α levels was also observed following PHS pretreatment (*P* < 0.001). These findings indicate that PHS effectively attenuates LPS-mediated inflammatory responses and may represent a promising therapeutic candidate for the modulation of inflammation associated with oral diseases.

## Discussion

The present study demonstrates that PHS effectively inhibits both planktonic growth and dual-species biofilm formation of *S. mutans* and *A. actinomycetemcomitans*, two key bacterial species associated with dental caries and aggressive periodontitis (AgP). Extensive evidence indicates that biofilm formation underlies the pathogenesis of dental caries and periodontitis, particularly in root caries and periodontal pocket sites^10^. Previous studies have reported increased biofilm biomass in dual-species biofilms involving *S. mutans* and *A. actinomycetemcomitans*^11^, highlighting the clinical relevance of targeting multispecies biofilm interactions and eradications.

In the present study, *S. mutans* formed large, densely clustered microcolonies characterized by prominent “cap-and-stalk”–like structures in single-species biofilms, consistent with previous observations^25^. Notably, these architectural features were preserved in dual-species biofilms with *A. actinomycetemcomitans*, indicating that biofilm structural development is predominantly driven by *S. mutans*. This supports its role as a primary biofilm architect, providing a structural scaffold that may facilitate the incorporation and persistence of *A. actinomycetemcomitans* within the biofilm ecosystem. This observation further supports the hypothesis that *S. mutans* promotes the accumulation of microorganisms on the tooth surface, thereby contributing to the establishment and maturation of pathogenic oral biofilms^3^.

Oral biofilms are dynamic microbial communities in which cooperative and competitive interactions regulate microbiome stability and influence the transition between health and disease^1^. In this study, a reduction in biofilm biomass of *S. mutans* single-species biofilms was observed at 48 h compared with 24 h, consistent with the natural biofilm dispersal behavior of this organism^26^. Notably, the presence of *A. actinomycetemcomitans* in dual-species cultures further reduced biofilm biomass at both time points. Previous studies indicate that *A. actinomycetemcomitans* exhibits reduced viability under mildly acidic conditions, which may be influenced by carbohydrate availability^27^. This acidification may partially contribute to the observed interspecies dynamics, particularly the reduced abundance or activity of *A. actinomycetemcomitans* in the present study. In contrast, *A. actinomycetemcomitans* exhibited increased biofilm formation at 48 h relative to 24 h, in agreement with previous reports^11^. However, these in vitro conditions may not fully reflect the complexity of the oral environment. Therefore, further studies using more physiologically relevant models are needed to better understand these dynamic microbial interactions. Furthermore, quantification of bacterial 16S rRNA expression in dual-species biofilms revealed significantly lower metabolic activity of *S. mutans* compared with *A. actinomycetemcomitans*, indicating a dominant *A. actinomycetemcomitans* population under coculture conditions. This may contribute to suppression of *S. mutans* growth, contributing to the observed reduction in dual-species biofilm biomass. Supporting this concept, *A. actinomycetemcomitans* has been shown to produce quorum-sensing molecules such as autoinducer-2 that inhibit biofilm formation by *Candida albicans*^28^ and its presence in saliva has been associated with reduced *S. mutans* growth^13^.

Studies have reported a positive association between *S. mutans* and periodontal pathogens such as *Fusobacterium nucleatum* in co-aggregation within dental biofilms^29^. In contrast, *Porphyromonas gingivalis* and *Treponema denticola* have been shown to interfere with the quorum-sensing system of *S. mutans*, reducing its biofilm formation and acid tolerance^30^. However, interactions between *A. actinomycetemcomitans* and *S. mutans* remain poorly understood. In this study, comparative proteomic analysis demonstrated that dual-species biofilms exhibited increased glycosyltransferase-mediated extracellular matrix production (GtfC) compared with monospecies *S. mutans* biofilms. These enzymes synthesize water-insoluble glucans that are essential for dental biofilm structure and stability^3,31^. This suggests that *A. actinomycetemcomitans* may enhance biofilm matrix formation by *S. mutans*, consistent with previous reports showing quorum-sensing regulon upregulation and increased biofilm formation in dual-species cultures^11^. In contrast, proteins associated with nutrient uptake (ABC transporters) and translation (the small ribosomal subunit protein bS16, trigger factor and elongation factor Ts) were downregulated, suggesting suppression of metabolic activity in *S. mutans*. Together, these findings indicate that *A. actinomycetemcomitans* differentially modulates *S. mutans* physiology within dual-species biofilms by enhancing biofilm structure and stability while concurrently suppressing nutrient uptake and translational activity in *S. mutans*. Nevertheless, the complex interactions within mixed-species biofilms require further investigation. However, this study provides the first proteomic evidence of an asymmetric competitive interaction in dual-species oral biofilms, where *A. actinomycetemcomitans* undergoes metabolic adaptation while *S. mutans* exhibits global metabolic suppression coupled with a stress-induced biofilm reinforcement response.

Patients with AgP have been reported to exhibit induction of the *glyA* gene, which encodes serine hydroxymethyltransferase (SHMT), in dental plaque samples, indicating an adaptive response to metabolic stress^32^. Our findings are consistent with these observations, as this enzyme was also overexpressed in dual-species biofilms. In addition, glyceraldehyde-3-phosphate dehydrogenase (GAPDH), which is commonly upregulated in periodontal pocket tissues and gingival crevicular fluid (GCF) from patients with periodontitis^33^, was similarly overexpressed in the dual-species culture. Conversely, the presence of *A. actinomycetemcomitans* was associated with reduced expression of ribosomal protein uS12 methylthiotransferase (RimO), an enzyme involved in post-translational methylthiolation^34^; however, its role in periodontal pathogenesis remains unclear. Taken together, these results suggest that dual-species biofilm formation promotes metabolic adaptation while selectively altering translational regulation in *S. mutans*, potentially contributing to microbial persistence and disease progression in periodontal environments. Although the proteomic and gene expression data suggest competitive interactions between *A. actinomycetemcomitans* and *S. mutans*, environmental factors associated with the in vitro culture system, including pH fluctuations, CO₂ levels, and nutrient depletion, may also have influenced bacterial abundance and biofilm development. Therefore, further studies using more physiologically relevant models are required to clarify the relative contributions of microbial interactions and environmental factors.

Mixed-species biofilms produce increased amounts of EPS, forming a dense and protective barrier that limits anti-microbial penetration^35^. PHS, a naturally occurring bioactive sphingolipid, has been reported to exhibit anti-microbial activity against *S. mutans*^19,22^, *P. gingivalis*^23^, and *C. albicans*^20^, and various pathogenic bacteria^21^. However, the effects of PHS on mixed-species biofilms have not previously been examined. In the present study, PHS effectively eradicated both planktonic and biofilm forms in single- and dual-species cultures. In planktonic co-cultures, conventional antibiotics (AMX and DOX) showed a slight reduction in bactericidal efficacy (MBC values), consistent with previous reports^4^. In contrast, PHS exhibited bactericidal activity at lower concentrations, achieving near-complete killing of planktonic bacteria at 5 µg/ml within 15 min. Biofilm-associated bacteria are known to exhibit 100- to 10,000-fold greater resistance than planktonic cells due to the EPS matrix, enhanced genetic exchange, and reduced metabolic activity^15,16^. Notably, PHS showed strong killing activity against biofilm-embedded bacteria in both single- and dual-species biofilms. This activity is attributed to the cationic head group of PHS, which interacts with negatively charged bacterial membrane components, leading to membrane destabilization, increased permeability, and cell death^22–24^. Collectively, these findings suggest that PHS exerts bactericidal activity against biofilm-associated persister or dormant cells, which are typically tolerant to conventional antibiotics requiring metabolically active targets^16^.

The urgent need to eradicate oral biofilms arises from their central role in the development of prevalent chronic infectious diseases, including dental caries and periodontitis, which are also associated with systemic health complications^36^. In this study, PHS exhibited strong anti-adherence activity against both single- and dual-species biofilms, consistent with previous reports^19,22^. Moreover, PHS effectively disrupted the biofilm matrix under all tested conditions. Although the precise anti-biofilm mechanism of PHS remains unclear, inhibition of amino acid uptake^37^ and induction of caspase-independent apoptosis have been reported^38^. A possible mechanism is that PHS interacts with the bacterial cell membrane, compromising membrane integrity and disrupting intracellular signaling, which leads to cytoplasmic leakage, cell death, and subsequent inhibition of biofilm maturation.

Salivary IL-6 and TNF-α levels are significantly elevated in patients with periodontitis compared with healthy individuals and serve as key pro-inflammatory biomarkers associated with tissue destruction and alveolar bone resorption^39^. IL-6 promotes alveolar bone loss by directly enhancing osteoclastogenesis through Th17-mediated responses^40,41^. In addition, bacterial endotoxins such as lipopolysaccharide (LPS) induce TNF-α via activation of NF-κB and mitogen-activated protein kinase (MAPK) signaling pathways, thereby stimulating osteoclast differentiation^42,43^. In this study, pretreatment with PHS significantly reduced IL-6 and TNF-α production induced by *P. gingivalis*-derived LPS in human gingival fibroblasts. Consistent with previous reports, the anti-inflammatory effects of PHS are associated with inhibition of NF-κB signaling, suppression of oxidative stress, and upregulation of PPARγ expression^44^. These findings suggest that PHS modulates host inflammatory responses and may have potential as a preventive or adjunctive therapeutic strategy for reducing periodontal inflammation.

The cytotoxicity results further support the clinical potential of PHS as a biocompatible anti-microbial agent. PHS demonstrated significantly higher cell viability than CHX after 24 h of incubation with HGF-1 cells. Minimal cytotoxicity was observed at 5 µg/ml PHS, a concentration sufficient to achieve bactericidal activity in planktonic cultures and to eradicate the biofilm matrix in dual-species biofilms. In contrast, CHX caused a marked reduction in cell viability, consistent with its well-documented cytotoxic effects^45^. Collectively, these findings indicate that PHS exhibits favorable biocompatibility and may represent a biocompatible candidate for further investigation.

## Methods

### Preparation of phytosphingosine (PHS)

The stock solution of PHS was prepared in 99% (v/v) ethanol at a concentration of 5 mg/ml, as previously described^46^. PHS was then diluted in a 20 mM Tris buffer (pH 6.8) supplemented with 0.1% Tween 20 (Tris-Tween) to achieve the desired concentration.

### Bacterial strain and growth condition

*S. mutans* ATCC 25175™ (ATCC^®^, Manassas, VA) and *A. actinomycetemcomitans* ATCC 43718™ (ATCC^®^) were initially cultured on Brain Heart Infusion (BHI) agar (HiMedia^®^, Mumbai, India) at 37 °C in 5% CO₂ for 24 h. A single colony from each strain was subsequently inoculated into BHI broth and incubated anaerobically at 37 °C in 5% CO₂ for 18 h. For biofilm formation, bacterial cultures were grown in BHI supplemented with 2% sucrose under the same incubation conditions^47^. Dual-species biofilms and planktonic cultures were established using a standardized 1:1 ratio of the two strains to ensure comparable initial bacterial loads.

### Antimicrobial susceptibility testing

The MIC and MBC of conventional antibiotics were determined using the broth microdilution method. Briefly, bacterial suspensions were adjusted to a final concentration of 5 × 10⁵ CFU/ml. Two-fold serial dilutions of DOX, AMX (Sigma-Aldrich, St. Louis, MO), and PHS were prepared in Mueller–Hinton broth (MHB) (HiMedia^®^) in 96-well microtiter plates (Nunclon™, Roskilde, Denmark) to achieve final concentrations ranging from 0.06 to 128 µg/ml. The plates were incubated at 37 °C in a 5% CO₂ atmosphere for 24 h. Wells containing bacterial cultures with culture medium and vehicle (PHS-free) served as growth controls and negative controls, respectively.

The MIC was defined as the lowest concentration of each agent that inhibited visible bacterial growth and was confirmed spectrophotometrically by measuring optical density at 620 nm (OD₆₂₀). The MBC was determined as the lowest concentration that resulted in no detectable colony growth on MHA after 24 h of incubation. The experiment was performed independently three times, each in duplicate.

### Time-kill assay of PHS against planktonic cultures

The antibacterial activity of PHS against planktonic cultures of single- and dual-species was evaluated as previously described^48^. Briefly, bacterial suspensions were adjusted to a concentration of 5 × 10⁵ CFU/ml and mixed with an equal volume of PHS to obtain final concentrations ranging from 0.31 to 10 µg/ml, covering concentrations below and above the MIC values and enabling evaluation of both inhibitory and biofilm-disruptive effects. The mixtures were incubated at 37 °C in a 5% CO₂ atmosphere for 15, 30, and 60 min. At each time point, samples were serially diluted, plated on BHI agar, and incubated for 24 h. Bacterial suspensions without PHS served as untreated controls. The experiment was performed independently three times, each in duplicate.

### Biofilm-forming capacity

The biofilm-forming ability of single- and dual-species biofilms was evaluated using a crystal violet staining assay as previously described^49^. Briefly, bacterial suspensions were adjusted to a concentration of 1 × 10⁸ CFU/ml and inoculated into sterile 96-well flat-bottom plates (Nunclon™). The plates were incubated at 37 °C in a 5% CO₂ atmosphere for 3 h to allow initial bacterial adhesion. Non-adherent cells were then removed and replaced with fresh medium, followed by further incubation for 24–48 h to allow biofilm maturation. After incubation, the biofilms were gently washed with sterile distilled water and fixed with 99% (v/v) methanol. Following air-drying, the biofilms were stained with 2% (w/v) crystal violet for 5 min. Excess stain was removed, and the bound dye was solubilized with 33% (v/v) glacial acetic acid. Biofilm biomass was quantified by measuring absorbance at 620 nm using a microplate reader. Each experiment was independently performed three times, with eight replicates per condition.

### Structure of bacterial biofilm

Bacterial aggregation during biofilm formation was observed using scanning electron microscopy (SEM) as previously described^50^. Briefly, 1.5 ml of overnight bacterial cultures (1 × 10⁸ CFU/ml) were added to 24-well tissue culture plates (Nunclon™) with glass coverslips (22 × 22 mm) and incubated at 37 °C in a 5% CO₂ atmosphere for 24 h. Biofilms were rinsed three times with 0.1 M cacodylate buffer and fixed with 3% glutaraldehyde for 1 h, followed with 1% osmium tetroxide for 1 h. The samples were then dehydrated through a graded ethanol series (30–100%) and dried under nitrogen gas before sputter-coating with gold–palladium. The biofilm structures were subsequently examined using SEM (JSM-6610LV, JEOL, Tokyo, Japan).

### Bacterial proportion

The bacterial proportions in single- and dual-species biofilms were quantified using qRT-PCR^51^. Briefly, bacterial suspensions (1 × 10^8^ CFU/ml) were inoculated into 24-well tissue culture plates (Nunclon™) and incubated at 37 °C in 5% CO₂ for 3 h to allow attachment. Non-adherent cells were removed and replaced with fresh medium, followed by incubation for an additional 21 h. Biofilms were scraped and transferred into wells containing RNAprotect Bacterial Reagent (Qiagen, Germantown, MD). Samples were centrifuged at 12,000 × g for 10 min, and the supernatant was discarded. Total RNA was extracted using the TRIzol™ Max™ Bacterial RNA Isolation Kit (Thermo Fisher Scientific, Waltham, MA). Residual genomic DNA was removed twice using TURBO DNA-free™ DNase Treatment (Ambion^®^, Carlsbad, CA). DNase-treated RNA was reverse transcribed into cDNA using iScript™ Reverse Transcription Supermix (Bio-Rad, Hercules, CA) in a thermal cycler (Applied Biosystems, Thermo Fisher Scientific). Gene expression of 16 S rRNA (Table 4) was quantified using PowerUp SYBR Green Master Mix on a QuantStudio™ 3 Real-Time PCR System (Thermo Fisher Scientific). The experiment was performed independently three times, each in duplicate.

**Table 4:** Primers used in this study.

Bacterial strain	Forward primer (5′−3′)	Reverse primer (5′−3′)
*S. mutans* ^52^	AGCGTTGTCCGGATTTATTG	CTACGCATTTCACCGCTACA
*A. actinomycetemcomitans* ^53^	GAA CCT TAC CTA CTC TTG ACA TCC GAA	TGC AGC ACC TGT CTC AAA GC

### Proteomic analysis

#### Sample preparation

The analysis of bacterial protein expression in single- and dual-species biofilms was performed as previously described with minor modifications^54^. Briefly, bacterial suspensions (1 × 10⁸ CFU/ml) were inoculated into 24-well tissue culture plates (Nunclon™) containing BHI supplemented with 2% sucrose and incubated at 37 °C under 5% CO₂ for 3 h to allow initial bacterial attachment. Non-adherent cells were then removed and replaced with fresh medium, followed by further incubation for 21 h. After 24 h, biofilms were harvested by scraping, and the resulting biofilm pellets were collected by centrifugation at 14,000 × g for 15 min prior to protein extraction. Three biological replicates of bacterial cell lysates obtained from the biofilms were resuspended in SP3 (Single-Pot, Solid-Phase-Enhanced Sample Preparation) lysis buffer containing 8 M urea in 100 mM triethylammonium bicarbonate (TEAB) supplemented with 1× protease inhibitor. Samples were incubated at 95 °C for 5 min, followed by incubation at 70 °C for 25 min. Proteins were digested using trypsin/Lys-C at a 1:50 (w/w) enzyme-to-protein ratio and incubated overnight at 37 °C. Peptides were collected by centrifugation at 14,000 × g for 20 min and desalted using C18 Jupiter desalting columns with reverse-phase Finisterre SPE C18 cartridges (WICOM International AG, Chur, Switzerland) to remove salts and impurities. Protein concentrations of the lysates were determined using a Pierce™ BCA Protein Assay Kit (Thermo Fisher Scientific). The experiment was performed independently three times.

#### LC–MS/MS analysis

Tryptic peptides obtained from the biofilm lysates were analyzed using a Q Exactive™ Plus Hybrid Quadrupole-Orbitrap™ Mass Spectrometer (Thermo Fisher Scientific) coupled to a Vanquish™ Neo UHPLC System (Thermo Fisher Scientific). Peptides were separated on a 50 cm EASY-Spray C18 analytical column (75 μm internal diameter) using a linear gradient of mobile phase A (0.1% formic acid in LC-MS grade water) and mobile phase B (0.1% formic acid in LC-MS grade acetonitrile) at a flow rate of 0.3 µl/min. The electrospray ionization voltage was set to 2.0 kV. Full MS scans were acquired over an m/z range of 350–1,400 at a resolution of 70,000, with an automatic gain control (AGC) target of 3 × 10⁶ ions and a maximum injection time of 250 ms. Data-dependent MS/MS acquisition was performed at a resolution of 17,500 with an AGC target of 5 × 10⁴ ions, a maximum injection time of 100 ms, and a loop count of 10. Precursor ions were isolated using a 1.2 m/z isolation window and fragmented by higher-energy collisional dissociation (HCD) with a normalized collision energy (NCE) of 27.

#### Protein identification

Raw mass spectrometry data were processed using Proteome Discoverer version 2.1.1.21 (Thermo Fisher Scientific). Peptide spectra were searched against the *S. mutans* UniProt database (2025 release), the *A. actinomycetemcomitans* UniProt database (2025 release), and a common contaminant database obtained from the Global Proteome Machine. Database searches were performed using trypsin as the proteolytic enzyme, allowing a maximum of two missed cleavages. Carbamidomethylation of cysteine (+ 57.02146 Da) was specified as a fixed modification, while methionine oxidation was included as a variable modification. The precursor ion mass tolerance was set to 10 ppm and the fragment ion mass tolerance to 0.02 Da. Peptide and protein identifications were filtered using a false discovery rate (FDR) of 1%, determined based on q-values. To ensure high-confidence protein identification, LC–MS/MS analyses were performed using a Q Exactive™ Plus system equipped with Advanced Quadrupole Technology (AQT) and maintained under routine preventive maintenance according to the manufacturer’s recommendations.

#### Data analysis

Protein abundance was quantified based on peptide peak areas. Only peptides identified with high confidence (q-value < 0.01) were included in the analysis. Intensity values were normalized using median normalization followed by log₂ transformation. Relative protein expression levels of proteins detected in both conditions were compared between single-species and dual-species biofilms. Proteins with an absolute log₂ fold-change ≥ 1.2 were considered up- or down-regulated. Enrichment analysis of differentially expressed proteins was performed using the STRING database (version 12.0), with protein accessions matched against *S. mutans* UA159 or *A. actinomycetemcomitans*. Enriched terms were considered significant at a false discovery rate (FDR) of < 0.05.

### Anti-bacterial adhesion

The anti-adhesive activity of PHS against bacterial attachment was evaluated using the static Amsterdam Active Attachment (AAA) model, as previously described with minor modifications^55,56^. Briefly, 1.5 ml of overnight bacterial cultures (1 × 10⁸ CFU/ml) were added to 24-well tissue culture plates (Nunclon™) in the presence of PHS at 5, or 10 µg/ml. The plates were covered with a sterile stainless-steel lid fitted with glass coverslips (22 × 22 mm) and incubated at 37 °C under 5% CO₂ for 3 h to allow initial bacterial attachment. Wells containing culture medium alone served as untreated controls. Following incubation, the coverslips were gently washed twice with sterile distilled water, and attached cells were stained with SYTO™ Green Fluorescent Nucleic Acid Stain (Invitrogen, Carlsbad, CA) to visualize viable bacteria. The sessile cells were imaged in two dimensions at the top, middle, and bottom regions of each coverslip using a confocal laser-scanning microscope (CLSM; Leica DMi8, Wetzlar, Germany). Fluorescence intensity ratios between PHS-treated and untreated samples were quantified using Leica Application Suite X (LAS X) software. The experiment was performed independently three times, each in duplicate.

### Effect of PHS on biofilm formation

The reduction of biofilm formation in single- and dual-species biofilms in the presence of PHS was evaluated as previously described with minor modifications^57,58^. Briefly, bacterial suspensions (1 × 10^8^ CFU/ml) were prepared in duplicate plates for biofilm quantification and cell viability assays. To assess interference with early biofilm development, bacterial suspensions were incubated with PHS (0.31–10 µg/ml) at 37 °C under 5% CO₂ for 24 h. After incubation, remaining biofilms were quantified using the crystal violet staining assay, and the percentage reduction in biofilm biomass was calculated as [1 − (OD₆₂₀ of treated/OD₆₂₀ of untreated)] x 100%.

In parallel, bacterial suspensions in another plate were incubated with PHS at the same concentrations for 24 h at 37 °C under 5% CO₂. Biofilm metabolic activity, as an indicator of cell viability following PHS treatment, was assessed by washing biofilm-embedded cells twice with sterile distilled water and incubating with MTT solution (0.5 mg/ml) for 4 h at 37 °C under 5% CO₂ according to the manufacturer’s instructions (Sigma-Aldrich). The experiment was performed independently three times, each in duplicate.

## Effect of PHS on biofilm matrix

The influence of PHS on the biofilm matrix was examined using AAA model, following a previously established protocol^59^. Briefly, overnight bacterial cultures (1 × 10^8^ CFU/ml) were inoculated into 24-well tissue culture plates (Nunclon™), which were then covered with a sterile stainless-steel lid fitted with glass coverslips. Biofilms were allowed to develop at 37 °C under 5% CO₂ for 24 h in the presence of 5–10 µg/ml PHS, while untreated wells served as controls. After incubation, the biofilm-coated coverslips were gently rinsed twice with sterile distilled water and fixed with 2.5% glutaraldehyde at room temperature for 1 h. The fixed biofilms were subsequently stained with FITC–ConA (50 µg/ml) for 30 min to visualize the exopolysaccharide matrix. Biofilm architecture was analyzed using CLSM (Leica DMi8), with images captured at multiple depths (top, middle, and bottom) and Z-stacks acquired at 0.5 μm intervals across the biofilm thickness. Quantitative assessment of biofilm matrix reduction was performed using COMSTAT software (BioCentrum-DTU, Lyngby, Denmark). The experiment was performed independently three times, each in duplicate.

### Cytotoxicity testing

HGF-1 cells (CRL-2021™, ATCC^®^) cells were cultured in Dulbecco’s Modified Eagle’s Medium (DMEM; HyClone™, Marlborough, MA) supplemented with 10% fetal bovine serum (FBS) (HyClone™) and maintained at 37 °C in a humidified atmosphere containing 5% CO₂ for 18 h. Cells were subcultured using standard procedures with 0.25% trypsin and 0.1% EDTA (Corning™, Corning, NY).

The cytotoxicity of PHS on HGF-1 was evaluated as previously described^60^. Briefly, cells were seeded at a density of 1.0 × 10^4^ cells/well in 96-well plates and grown overnight. Cells were exposed to 0.08–10 µg/ml PHS and 0.002–0.2% CHX for 24 h at 37 °C in a 5% CO_2_ atmosphere. Cells cultured in medium alone served as untreated controls. Cytotoxicity was assessed using the MTT assay (0.5 mg/ml) according to the manufacturer’s instructions (Sigma-Aldrich). The experiment was performed independently three times, each in duplicate.

### Anti-inflammatory cytokine of PHS on LPS-induced cells

The effect of PHS on the reduction of pro-inflammatory cytokine secretion was evaluated as previously described with minor modifications^61,62^. HGF-1 were seeded at a density of 1 × 10⁵ cells per well in 12-well culture plates and cultured overnight. Cells were pretreated with 5 µg/ml PHS for 1 h at 37 °C in a humidified atmosphere containing 5% CO₂. This concentration was selected based on its minimal cytotoxic effects on HGF-1 cells and its ability to effectively disrupt the biofilm matrix. Subsequently, the cells were stimulated with 5 µg/ml LPS from *P. gingivalis* (Sigma-Aldrich) for 24 h. Cells treated with LPS alone without PHS served as positive controls, whereas cells cultured in medium alone served as negative controls.

After incubation, culture supernatants were collected and centrifuged at 2,000 × g for 10 min to remove cellular debris. The supernatants were diluted 1:10, and the concentrations of IL-6 and TNF-α were quantified using ELISA kits according to the manufacturer’s instructions (Abcam^®^, Cambridge, UK). The experiment was performed independently three times, each in duplicate.

### Statistical analysis

Data are presented as mean ± standard deviation (SD). An independent t-test was used to analyze the relative abundance of 16S rRNA in dual-species biofilms. Comparisons of biofilm biomass and cytokine expression were performed using one-way ANOVA followed by Tukey’s multiple-comparisons test. Biofilm-forming capacity and cytotoxicity were analyzed using two-way ANOVA followed by Tukey’s and Sidak’s multiple-comparisons tests, respectively. All statistical analyses were performed using GraphPad Prism software (version 9).

## Conclusions

This study demonstrates that PHS exhibits in vitro anti-microbial and anti-biofilm activity against *S. mutans* and *A. actinomycetemcomitans* in both single- and dual-species models, effectively eradicating planktonic cells and disrupting the biofilm matrix. PHS pretreatment significantly reduced LPS-induced IL-6 and TNF-α production in human gingival fibroblasts, indicating a role in modulating host inflammatory responses while exhibiting minimal cytotoxicity. Collectively, these findings indicate that PHS may serve as a potential dual-function agent with antimicrobial and anti-inflammatory properties. However, further studies, including in vivo and clinical investigations, are required to confirm its therapeutic applicability in biofilm-associated oral diseases.

## Supplementary Information

Below is the link to the electronic supplementary material.

Supplementary Material 1.

## Data Availability

All data generated or analysed during this study are included in this published article and its supplementary information files.
